# Topological Maps and Brain Computations From Low to High

**DOI:** 10.3389/fnsys.2022.787737

**Published:** 2022-05-27

**Authors:** Martin I. Sereno, Mariam Reeny Sood, Ruey-Song Huang

**Affiliations:** ^1^Department of Psychology, San Diego State University, San Diego, CA, United States; ^2^Department of Psychological Sciences, Birkbeck, University of London, London, United Kingdom; ^3^Centre for Cognitive and Brain Sciences, University of Macau, Macau, Macao SAR, China

**Keywords:** extrastriate cortex, retinotopy, tonotopy, somatotopy, cortical computation, serial assembly of content

## Abstract

We first briefly summarize data from microelectrode studies on visual maps in non-human primates and other mammals, and characterize differences among the features of the approximately topological maps in the three main sensory modalities. We then explore the almost 50% of human neocortex that contains straightforward topological visual, auditory, and somatomotor maps by presenting a new parcellation as well as a movie atlas of cortical area maps on the FreeSurfer average surface, *fsaverage*. Third, we review data on moveable map phenomena as well as a recent study showing that cortical activity during sensorimotor actions may involve spatially locally coherent traveling wave and bump activity. Finally, by analogy with remapping phenomena and sensorimotor activity, we speculate briefly on the testable possibility that coherent localized spatial activity patterns might be able to ‘escape’ from topologically mapped cortex during ‘serial assembly of content’ operations such as scene and language comprehension, to form composite ‘molecular’ patterns that can move across some cortical areas and possibly return to topologically mapped cortex to generate motor output there.

## Introduction

There is a long history of trying to compactly characterize the canonical computational principles of cerebral cortex (and other) areas in the brain. Given the incredibly diverse nature of sensory information arriving from different sensory modalities as well as the diverse geometry of motor output structures (e.g., eye muscles versus arm muscles), this might seem at first too quixotic a quest.

**FIGURE 1:**
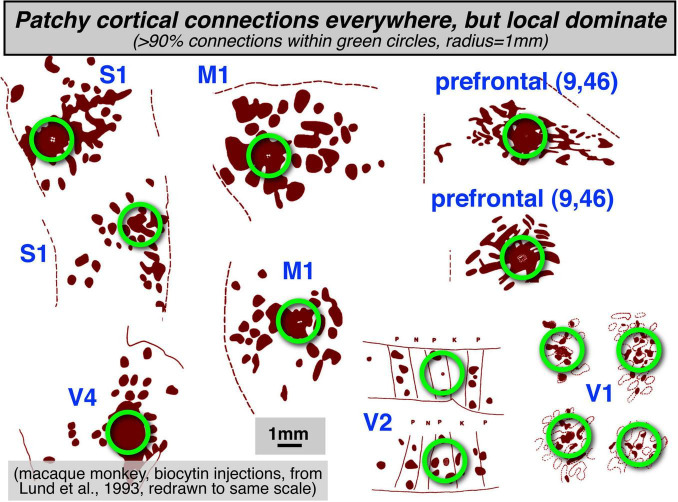
Patchy local structures and connections are found everywhere in the cortex. However, the numerical majority of connections are made within a 1 mm radius (green circles).

Two prominent features that are found throughout much of the cerebral cortex are: (1) a strong predominance of extremely local connections, and (2) longer-range interareal connections that form topological maps. Though visually attractive patchy local connections (e.g., innervating adjacent cytochrome oxidase blobs) are often highlighted in neuroanatomical and modeling studies, more local connections (within a 1 mm radius) are strongly numerically dominant throughout the cortex (see Figure 1, redrawn from multiple figures in Lund et al., 1993, with different cortical regions all set to the same scale). Zooming out to an intermediate scale, longer-range connections between areas are then most commonly arranged as approximately topological (neighbor-preserving) maps, initially maps of sensory surfaces, and then at the output, maps of muscle arrays or effector output space. As sensory and motor information is passed from station to station, topological maps remain an extremely common motif.

We don’t want to downplay the beautifully and complexly intercalated maps of different local stimulus features such as orientation (interblobs), and brightness and color (blobs) that have been studied in the greatest detail in area V1 in cats and primates (e.g., Swindale et al., 2000; Carreira-Perpin and Goodhill, 2004; Sincich and Horton, 2005; Yu et al., 2005). This pattern of embedding multiple streams of information that emphasize different features within an overall topological map is ubiquitous throughout the visual (and auditory and somatosensory) systems. Higher level examples from the visual system include subdivisions within V2 stripes (Wang et al., 2007; Lim et al., 2009), MT direction columns and band/interbands that respect or ignore background motion (Albright, 1984; Born and Tootell, 1992; Diogo et al., 2020). For the present purposes, it is merely that we have zoomed out to concentrate on the retinotopic (and tonotopic and somatotopic) maps.

This topological, neighbor-preserving mode of intermediate scale neural interconnection is surprisingly ubiquitous throughout the brain. Approximately topological map connections extend not only to thalamic structures projecting to and receiving inputs from visual, auditory, and somatosensory cortical areas, but are seen in many other structures not as well known for containing topological maps.

Here are just a handful of hundreds of possible examples: (1) The projections from the caudate and putamen to both components of the substantia nigra (GABAergic pars reticulata and dopaminergic pars compacta) are about as topological as the projection from V1 to V2 (Parent and Hazrati, 1994). (2) There are approximately topological connections between many cortical areas and the subthalamic nucleus (the ‘hyperdirect’ pathway; Haynes and Haber, 2013). (3) Odor-specific olfactory receptors in the olfactory epithelium sort themselves into a spatial odor map in their projection to the olfactory bulb (Nakashima et al., 2021), and then topological intrabulbar (left-right and right-left) projections respect this odor map (Schoenfeld et al., 1985). (4) Topological sensory maps are found in a modified form in the large surface area of the cerebellar cortex (Sereno et al., 2020), within the small patches that make up the unique ‘fractured somatotopy’ mosaic found there (Shambes et al., 1978). (5) The cerebellar-connected inferior olive and the cerebellar dentate nucleus are each crumpled into miniature cortical surfaces embedded within the brainstem in order to preserve two-dimensional maps there (Ding et al., 2016, their pp. 215, 243).

Approximately topological maps are also common in output structures (e.g., the cerebellar dentate nucleus just mentioned). The spatial array of muscles controlled by motor cortex was in fact the first cortical topological brain maps uncovered experimentally (Fritsch and Hitzig, 1870). Another well-known topological map is found in the deeper layers of the superior colliculus – a map of saccade and neck movement vectors – which receives topological map connections from the retinotopic superficial superior colliculus (Sparks, 1986) and manipulates topological maps of auditory- and somatosensory-space targets by shifting them to keep them aligned with the retinotopic map after each saccade by using eye position information (e.g., Jay and Sparks, 1984). It could fairly be said that topological maps are the first principal component of mid-scale brain organization.

Despite their ubiquity, however, there has also been the intuition that areas containing maps must somehow *not* be appropriate for supporting higher level cognition (e.g., Pylyshyn, 1984; McClelland and Rumelhard, 1986). This is only the latest rehearsal of a several centuries-long debate between “field” and “localization” theories of cortical function. As a foil, there has been an equally long history of trying to mimic biological topological maps using artificial neural networks (e.g., Fukushima, 1980; Linsker, 1986; Sereno and Sereno, 1991). Although the implicit bias against using tiered topological maps in machine learning has been much relaxed in recent years with the popularity of ‘convolutional neural networks’ (LeCun et al., 2015), the intuition of many researchers that use human neuroimaging methods in cognitive neuroscience is still that most important ‘higher level’ areas in the cortex must *not* contain simple topological maps.

Several possibilities come to mind at this juncture. First, it may be true that ‘higher areas’ do in fact compute without maps of any kind (though certainly after being fed inputs from areas with maps), using distributed activity patterns that would appear on casual inspection to be spatially random. Second, higher areas might contain static spatial feature maps (e.g., Kohonen, 1982); but we might have only begun to divine their mostly unknown coordinates (this might be near to the consensus view). A third more speculative possibility explored here is that certain regions of the cortex might host coherent, spatially localized patterns of activity capable of moving across the cortex and bonding with each other while maintaining their internal spatial structure (Sereno, 1991, 2014); despite having coherent, spatially localized structure from moment to moment, these moveable activity patterns might be difficult to detect using standard, low temporal precision fMRI mapping methods.

We first briefly summarize data from microelectrode studies on visual maps in non-human primates and other animals, and describe how approximately topological maps differ among the three main sensory modalities. We then describe the almost 50% of human neocortex that does contain straightforward topological visual, auditory, and somatomotor maps and present a new downloadable cortical parcellation and atlas movie of those 117 areas in each hemisphere that is based on the FreeSurfer average surface, subject *fsaverage*. Third, we review recent data on moveable maps and then review a recent experiment suggesting that cortical activity during sensorimotor actions involves coherent traveling waves and bumps. Finally, we speculate briefly on the third possibility introduced above, that coherent localized spatial activity patterns might be able to ‘escape’ from mapped areas and move across cortical areas that do not contain straightforward sensory maps, by analogy with remapping phenomena, and that they may eventually re-enter topological maps on the way to motor output.

## Materials and Methods

### Microelectrode Retinotopic Mapping and Parcellation

To characterize retinotopic maps at a 100 micron scale, dense retinotopy data sets were obtained by microelectrode visual receptive field mapping in dorsal and lateral visual cortex of anesthetized owl monkeys and then parcellated using the visual field sign method, which measures the local relation between the cortical gradient in polar angle and the cortical gradient in eccentricity to distinguish mirror-image from non-mirror-image visual field representations (Sereno et al., 1994). Afterward, the neocortex was physically flatmounted and penetration photograph recording locations were warped into alignment with the flattened myelin-stained cortex using marker lesions and a deformable template algorithm (see Sereno et al., 2015, for details).

### fMRI Mapping Experiments

To comprehensively catalog topological maps in humans, phase-encoded retinotopic, tonotopic, and somatomotor-o-topic fMRI data (see Dick et al., 2012; Huang and Sereno, 2013, 2018; Sood and Sereno, 2016, 2018, for more details) were collected at 1.5T and 3T using the X11/OpenGL phase-encoded stimulus program, *mapper*.^1^ For visual experiments, a wide-field, direct-view in-bore screen (projection from the front) was used, which stimulated eccentricities to 50 deg at all polar angles. Auditory stimuli (bandpass filtered sweeps of emotional vocalizations taken from Dick et al. (2012) were presented through piezoelectric drivers (Sensimetrics). Somatomotor mapping was done by using brief auditory cues to subjects who carefully and minimally moved individual body parts in a repeated sequence. Quantitative T1-mapping data to map myelination from an overlapping set of subjects (Sereno et al., 2012) was also consulted.

However, to reduce blurring, the data directly used for drawing areal borders here was restricted to the set of experiments reported in Sood and Sereno (2016). That data was collected in the same set of subjects across the three different modalities, in a 1.5T scanner (Siemens Avanto), all using a 32-channel head coil (modified to remove the two eye coils in order to unblock the visual field). 80% of the fMRI data used the Minnesota Center for Magnetic Resonance Research multiband pulse sequence, with 4 simultaneously excited slices, no GRAPPA acceleration, a voxel size of 3.2 mm × 3.2 mm × 3.2 mm, a repetition time (TR) of 1 s, echo time (TE) of 54.8 ms, and 512 data volumes per scan, so each individual mapping scan was 8 min, 32 s. The initial 20% of the data used slightly thicker 3.8 mm slices and the unaccelerated Siemens product EPI sequence with TR = 2 s. Four scans were done for each subject for each modality for a typical total of 6,144 data volumes per subject. A T1-weighted alignment scan with the same block center and orientation was used to initialize the registration, which was then refined using FreeSurfer *bbregister*. No field maps were acquired, but spatial distortions at 1.5T in visual, auditory, and somatosensory areas were minimal.

Individual cortical surfaces were first reconstructed from the average of two T1-weighted scans (MP-RAGE, 1 mm × 1 mm × 1 mm, flip = 7 deg, TI = 1,000 ms, TR = 8.4 ms, TE = 3.57 ms) using FreeSurfer 5.3^2^ (Dale et al., 1999; Fischl et al., 1999a,b). Subsequent processing steps were performed using FreeSurfer-compatible *csurf*^3^, another extension of the core surface reconstruction tools introduced in Dale and Sereno (1993). Phase-encoded fMRI data sets were analyzed using Fourier-based methods (Engel et al., 1994; Sereno et al., 1995) and then computed 3D statistics were sampled to individual subject’s cortical surfaces along the surface normal to each vertex. Surface-based data were averaged across subjects using surface-based alignment driven by sulcal depth (FreeSurfer *mris_register*), masked by calculating a complex-valued F-ratio (Hagler et al., 2007, implemented in *csurf*), and then displayed on the FreeSurfer 40-subject average cortical surface (subject *fsaverage*) in unfolded and flattened views.

The improved *inflated_avg* surfaces (distorted triangles around north/south ‘poles’ repaired) and the new flattened surfaces (*cortex2.patch.flat*) made from them that were used here are included in the *csurf* distribution above. The *inflated_avg* surfaces are much less distorted than the more familiar *inflated* surface (and the flattenings made from it) in the standard FreeSurfer distribution. The standard *inflated* surface for subject *fsaverage* is made by surface-averaging the coordinates of the *orig* (folded) surfaces of individual subjects, and then inflating the result. By contrast, the fsaverage *inflated_avg* surface is made by first inflating the folded surface for each individual subject and then surface-averaging the coordinates of the already inflated surfaces. These subtly different processing streams (folded/average/inflate *versus* folded/inflate/average) result in markedly different outcomes as a result of the many idiosyncratic local crinkles in the major sulci of individual brains. When folded surface coordinates are surface-averaged, these crinkles are removed and average sulci are straightened. However, this process also selectively reduces the surface area of the sulci in the average surface; as a result, the total surface area of the average *inflated* surface is reduced by about 1/3 compared to a typical individual brain surface. By first removing the idiosyncratic sulcal crinkles by inflating individual subject surfaces, and then surface-averaging the individual *inflated* surfaces to produce the *inflated_avg* surface, the anisotropic surface shrinkage bias is removed. These less distorted inflated surfaces can then be flattened to give a more veridical flattened template.

Topological cortical maps were defined as contiguous groups of surface vertices with significant periodic response to phase-encoded mapping stimuli that included a range of response phases. For visual mapping, we averaged two counter-clockwise and two time-reversed clockwise rotating polar angle wedge scans, for auditory mapping, two ascending and two time-reversed descending bandpass-filtered non-verbal vocalization scans (cf. Rauschecker et al., 1995), and for somatomotor mapping, two face-to-foot and two time-reversed foot-to-face bilateral cued voluntary movement of individual body part scans (see Huang and Sereno, 2013, 2018; Sood and Sereno, 2016, 2018 for details). Time-reversed datasets were time-shifted 5 sec before being averaging with unreversed data to account for estimated average hemodynamic delays; the time-reversal cancels delay *differences* between different regions. After aligning individual subject spheres with the *fsaverage* sphere (FreeSurfer *mris_register*), data was sampled to the average space with one step of nearest neighbor surface smoothing (FreeSurfer *mri_surf2surf*). Average data was then smoothed with one additional nearest neighbor smoothing step for display. Together, that corresponds to a 2D FWHM kernel of only 1.4 mm (Hagler et al., 2007), substantially narrower than the 3.2 mm fMRI voxel width.

### Manual Parcellation of Cross-Subject Average fMRI Data

Single cortical area labels for the surface-averaged data for each modality were then manually generated using csurf *tksurfer* tools by cutting and surface-filling individual connected cortical surface patches for the left and right hemispheres that each contained a topological map representing most of each corresponding sensory receptor (hemi-) surface, respecting as best as possible the sometimes conflicting definitions of cortical areas from our three papers as well as a large number of similar papers from the literature (including, but not limited to: visual: Tootell et al., 1997; Press et al., 2001; Sereno et al., 2001; Wandell et al., 2005; Larsson and Heeger, 2006; Swisher et al., 2007; Caspers et al., 2008; Amano et al., 2009; Kolster et al., 2010; Pitzalis et al., 2010; Wandell and Winawer, 2011; Huang and Sereno, 2013, 2018; Wang et al., 2015; Glasser et al., 2016; Sood and Sereno, 2016; auditory: Pandya and Sanides, 1973; Kaas and Hackett, 2000; Talavage et al., 2004; Kusmierek and Rauschecker, 2009; Rauschecker, 2011; Striem-Amit et al., 2011; Dick et al., 2012; Moerel et al., 2014; Leaver and Rauschecker, 2016; Frank and Greenlee, 2018; Zeharia et al., 2019; somatomotor: Krubitzer et al., 1995; Padberg et al., 2007; Meier et al., 2008; Filimon et al., 2009; Seelke et al., 2011; Huang et al., 2012; Serra et al., 2019; general: Campbell, 1905; Brodmann, 1909; von Economo and Koskinas, 1925; Triarhou, 2007). Though we are familiar with the large mapping literature in nonhuman primates, carnivores, and other mammals, we could only superficially discuss it here [e.g., multiple ‘primary’ auditory areas were initially recognized in cats (Knight, 1977), but the relation to primates was obscured by the greatly reduced rotation of the temporal lobe in carnivores].

It is an unfortunate fact that picking any single name for a cortical region outside of V1/V2, 3b/1, and A1 will unavoidably conflict with many papers from many different laboratories. We attempted to use (or adapt) existing areal names with a preference for initial use in the literature. We constrained same-named areas in the left and right hemisphere to be in similar positions, with similar neighbors, and with similar orientations of the topological map gradient (gradient of the phase angle of the periodic response with respect to local tangential 2D cortical position). Arrow fields representing the gradient of map phase were computed and displayed using csurf tksurfer *compute_surf_grad* on both the folded or inflated surfaces to aid our manual parcellation (cf. Leaver and Rauschecker, 2016).

### Cortical Parcellation and Public Distribution

The collections of individual labels for each modality were then assembled into a parcellation of the entire neocortex of each hemisphere, and presented as FreeSurfer “annotation” files (rh-CsurfMaps1.annot, lh-CsurfMaps1.annot) for the right and left hemisphere FreeSurfer average surfaces (subject *fsaverage*). These parcellations define an area name and an area color for each vertex on the FreeSurfer average surfaces. They were assembled using csurf tksurfer *write_mgh_annot*, as directed by the color lookup table text file, CsurfColorLUT.txt. The FreeSurfer “annotation” files for each *fsaverage* hemisphere were also converted to GIFTI xml files (*.label.gii suffix) for use in other programs. Both annotation file types, the ASCII color table, and high resolution figure images are included in the *csurf* distribution above, and are also available for individual download here: https://cogsci.ucsd.edu/~sereno/csurf/fsaverage-labels/.

In more detail, the FreeSurfer annotation files specify an RGB color for each vertex on the left and right hemispheres of the FreeSurfer, *fsaverage*, followed by a color lookup table where each line lists a unique region RGB color, region name, and region ID number. The functionally equivalent GIFTI xml files begin with a color lookup table in the same format followed (more standardly) by a list of the integer region id numbers for each vertex that refer to the color lookup table. The FreeSurfer (or GIFTI) parcellation files can be used to sample ROIs from any data set that has been mapped onto the FreeSurfer average surface; in addition, the average surface cortical areas can be mapped back to an individual subject’s surface (e.g., using FreeSurfer *mri_surf2surf*) in order to pick out 2D regions of interest from an individual subject’s data. From there, the individual subject surface patches can also be used to pick out surface-normal-intersecting 3D-voxel-based gray matter ROI’s in subject-native fMRI space (e.g., using csurf tksurfer *annot2roi.tcl*). Finally, instructions for converting the GIFTI annotation files to the hemisphere independent (fs_LR) Human Connectome Project HCP sphere can be found here: https://wiki.humanconnectome.org/download/attachments/63078513/Resampling-FreeSurfer-HCP_5_8.pdf.

### Average Map Color Scales

Visual, auditory, and somatomotor maps are displayed with similar, easy-to-remember color scales: *green* for lower field, low frequency, or leg/foot; *blue* for horizontal meridian, mid frequency, or arm/hand, and *red* for upper field, high frequency, or face. Though more hues can be used to visually distinguish more levels of each map coordinate, more hues are also harder to keep in mind; and with more hues, small overall offsets in map coordinates can result in more distracting changes in visual appearance.

### Parcellation Philosophy

The goal of this exercise was to produce a tentative parcellation of contiguous areas based almost entirely on topological mapping data for the three main sensory modalities, while respecting approximate bilateral symmetry. By contrast, it has often been noted, by ourselves and others (Felleman and Van Essen, 1991; Sereno and Allman, 1991), that in the fullness of time, cortical areas are best defined by combining multiple features, which can include, for example, surface-based coordinates (e.g., after surface-based alignment driven by sulcus depth and/or other measures), topological sensorimotor map coordinates (what we are using here), functional connectivity measures, estimates of quantitative T1 values (Sereno et al., 2012; Glasser et al., 2016), diffusion surface (HARDI) features referenced to the local cortical surface normal (Nagy et al., 2013; Ganepola et al., 2018, 2021), responses from cognitive subtraction paradigms, effects of lesions, and so on. An obvious advantage of combining features for parcellation is that a border not detectable by one feature (e.g., T1 value) may be easily visualized when using a different feature (e.g., retinotopy).

However, one advantage of having parcellations primarily based on a single feature is that it is more straightforward for subsequent studies to assess which borders are robustly and independently localized by multiple features and which ones depend only on a single feature, and are therefore less robust. In addition, without maps of individual features (like the topological map boundaries here), it is more difficult to investigate cases where different map features disagree on the location of borders. One of our goals is to provide a resource that can be reused or revised in future multi-feature parcellations. Trying to keep parcellations editable, interchangeable, and combinable is a challenge given different software environments, but a worthy goal because cortical parcellations should best be viewed as works that are permanently in progress (Fischl and Sereno, 2018).

## Results

### Visual Areas in Non-human Primates Defined by Microelectrode Retinotopic Mapping

An extensive history of using microelectrode retinotopic mapping experiments to define visual areas in non-human primates and other animals has shown that large, early visual areas such as V1 and V2 can be relatively easily located and mapped. However, the difficulty of defining visual areas increases substantially with higher areas, which are invariably smaller, somewhat more variable across individuals and species, and which often contain partial representations of the visual hemifield. Perhaps the ‘next best’ cortical visual area to V1 and V2 in primates is area MT, which reliably contains a simple hemifield map, with 2/3 of its border marked by a clear change in myelination (Sereno et al., 2015, their Figure 3).

#### Parcellation by Visual Field Sign

**FIGURE 2:**
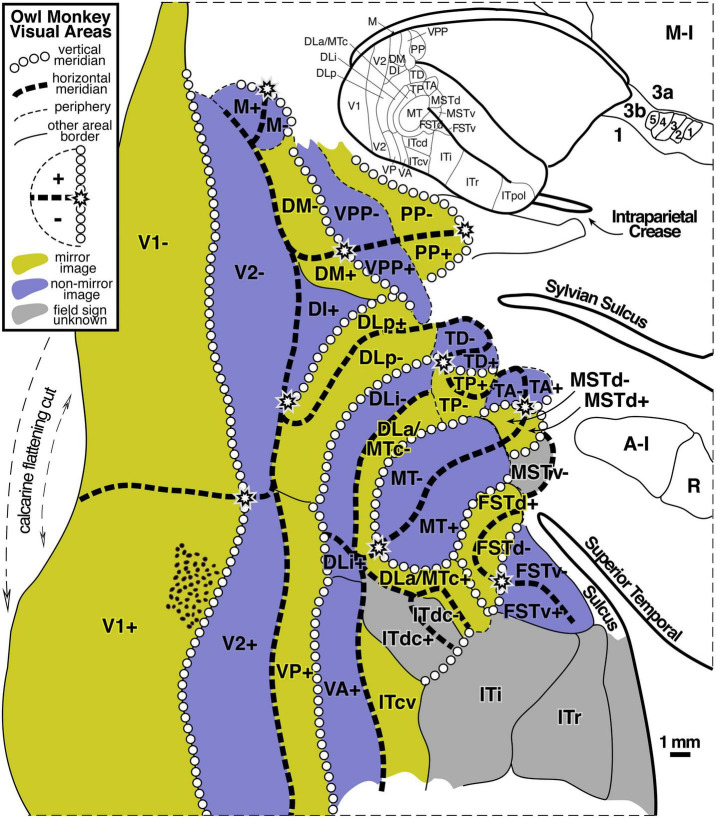
Owl monkey visual areas. Almost all of the 24 areas shown are retinotopic. Many contain partial representations of the visual field. Modified from Sereno et al. (2015).

By using high microelectrode penetration densities, it has been possible to demarcate a large number of additional visual areas beyond V1, V2, and MT. For example, Figure 2 (modified from Sereno et al., 2015) shows the retinotopic organization of dorsal and lateral visual areas in the owl monkey, using local visual field sign (mirror-image vs. non-mirror-image representation) to parcellate the data sets. Almost all of the visual areas in the owl monkey exhibit a substantial degree of retinotopic organization, which was apparently absent only in anterior inferotemporal cortex.

The large number of higher level areas combined with their small size (some containing full visual quadrant representations spanning barely more than 1 mm of cortex – see scale bar), has made it challenging to reliably locate and identify similar areas across individuals and across species, and some uncertainty about how to combine partial visual field representations remains. For a slightly different parcellation of visual areas in marmosets, another New World monkey, see Angelucci and Rosa (2015). For a visual field sign analysis of fMRI data from Old World macaque monkeys suggesting stronger than expected similarities between retinotopic map organization in New and Old World monkeys, see Janssens et al. (2014), Kolster et al. (2014), and Zhu and Vanduffel (2019).

#### Visual Receptive Field Position Is Continuous for Small Tangential Movements Across Cortex

These and many similar experiments have established that discontinuities in the cortical representation of visual space – defined as instances where a small movement tangential to the cortical surface in the recording location results in a discontinuous jump in the location of the corresponding visual receptive field – are extremely rare across the large expanse of extrastriate areas. In all but a very few instances, nearby recording sites (within 0.25 mm) have partially overlapping receptive fields (see Sereno et al., 2015, their Figure 9 for an extremely rare exception to this rule, and compare Yu et al., 2020, for an interesting proposal that a visual area in this region has a ‘twisted’ representation that contains adjoined mirror-image and non-mirror image parts, which would require a localized discontinuity at their border).

This finding also implies that the great majority of the borders between visual areas are ‘congruent,’ which means that adjoining areas virtually always have representations of similar (duplicated) visual field locations on either side of their shared border, the paradigm case being the vertical meridian border between V1 and V2 (Allman and Kaas, 1975).

#### Visual Maps Can Be Very Small

Experiments on mice (anatomical: Wang and Burkhalter, 2007; visual field sign analysis of intrinsic optical signals: Garrett et al., 2014), have revealed that their higher level visual areas can be truly tiny, with an entire representation of the visual hemifield crammed into a narrow column extending through the layers of the cortex that covers only 0.1 square mm of the cortical surface. Though it is not thought that visual areas in humans ever get this small, in part because human cortex is several times thicker than mouse cortex, the fact that complete visual areas *can* be so small suggests that we keep an open mind about what the minimum size of a cortical area map in humans is until positive data with high enough resolution has definitively set a lower size bound. In any case, the owl monkey data show that a complete visual quadrant representation can in many cases be narrower than a single standard 2–3 mm wide fMRI voxel (see area DLa/MTc in the middle of Figure 2).

### Differences Between Topological Maps in Different Modalities

As a preface to discussing topological maps in other modalities beyond vision, it is important to recognize several fundamental differences among the approximately topological maps in the visual system, the somatosensory system, and the auditory system.

#### Cortical Map Discontinuities (in Receptive Field Position as Function of Cortical Position)

The visual system moves a retina smoothly over objects in visual scenes. Because fixation points on scenes (and the content of scenes themselves) are so various, nearby points on the retina will tend to be activated across time in strongly correlated ways – that is, it is rare for object boundaries or other image discontinuities to repeatedly fall on the exact same retinal location so that one retinal region is repeatedly stimulated in a different way than a directly adjoining retinal region. This retinal stimulation pattern may explain – in the context of a correlation-based (Hebbian) topological map refinement rule (or other local substance-dependent learning rule) – the empirical observation noted above that short movements in recording location tangential to visual cortical areas very rarely result in discontinuous jumps in the location of the corresponding visual receptive field position.

By contrast, in the somatosensory system, it is trivial to generate stimuli that reliably respect a specific border between adjoining cortical regions. For example, one can easily stimulate the underside of the index finger while simultaneously completely avoiding stimulating the underside of the middle finger. Because the representations of the hairless (glabrous) undersurface of the five fingers are immediately adjacent to each other in area 3b, neurons on either side of the cortical border between adjacent fingers can reliably have uncorrelated responses; this is much less likely to reliably occur with any pair of nearby neurons in a visual cortical map.

A second difference between the visual and somatosensory system is that the retinal surface does not have anywhere near as much regionally-variable intrinsic (Gaussian) curvature as does the surface of the skin. This makes it less problematic to map the retinal surface to the cortical surface without introducing a large amount of local areal distortion. This is readily appreciated by imagining physically flattening a hemiretina, roughly a quarter of a sphere, which would hardly even require cuts. By contrast, trying to flatten the entire skin surface (including the surface of the hands, feet, lips and inside of the mouth, and so on) without introducing massive local areal distortion obviously requires many more cuts. Rather than a homunculus, somatosensory cortex actually consists of an unfamiliar patchwork quilt of skin regions with discontinuities at the borders between these patches. For example, the representation of the glabrous surfaces (hairless under surface) of all five fingers are neatly cut out and juxtaposed in area 3b, but then the representations of the hairy upper surfaces of the same fingers are separately cut out and placed off to the side (medially and laterally) from the glabrous surface array in area 3b (Merzenich et al., 1978).

These differences between visual and somatosensory receptive surfaces are likely responsible for the fact that discontinuities of the kind that only very rarely occur in the visual system are extremely common in the somatosensory system. For example, when traversing the cortical boundary between the index finger and the middle finger in area 3b, corresponding receptive fields discontinuously jump from being entirely on one finger to entirely on the other finger. There are hundreds of discontinuities of this type in early somatosensory maps, often arranged into lines across the cortical surface. For example, the representation of the underside of the thumb is directly apposed to a representation of a portion of the chin. Many of these discontinuities are visible in the cortex as thin lines of less dense myelination (Sereno, 2005, their Figure 1; Kuehn et al., 2017), probably reflecting reduced local cortical connectivity across these map discontinuities.

#### Converse Cortical Map Discontinuities (in Cortical Position as Function of Receptive Field Location)

Finally, it’s worth noting that the kind of discontinuity discussed above – involving jumps in receptive field location due to a small movement across a cortical map – should be distinguished from the converse kind of discontinuity – defined by a jump *on the cortex* resulting from a small movement *in the visual field*. Allman and Kaas (1975) called this converse kind of discontinuity a ‘second order transformation’ of the visual field. A well known example occurs at the anterior border of area V2, which represents the horizontal meridian. A small movement in the visual field from the lower visual field into the upper visual field that crosses the visual field horizontal meridian results in a sudden large jump in the corresponding location of the elicited cortical activity – from a point below the calcarine sulcus to a point above the calcarine sulcus. This converse kind of discontinuity is quite common in visual cortex, likely the end result of having to accommodate congruent borders between areas. Note that a congruent border (i.e., no discontinuity of the first kind) may be maintained at the location of this second, converse kind of discontinuity. For example, the border between lower field V2 and V3, which represents the horizontal meridian, is a congruent border between two quadrant representations (no discontinuity of the first kind), despite the fact that the horizontal meridian is the site of a discontinuity of the second, converse kind.

Similar converse discontinuities might occur in somatosensory and auditory areas. For example, is possible that some auditory areas may have an analogous V2-like split where the representation of lower frequencies is spatially detached in the cortex from the representation of higher frequencies.

#### 1D vs. 2D Sensory Surfaces

**FIGURE 3:**
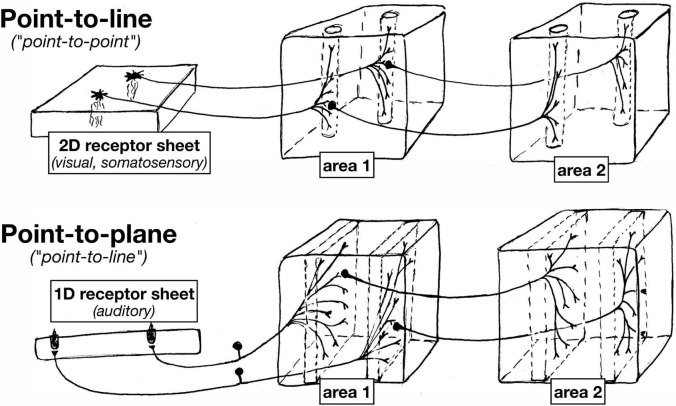
Since auditory receptors form a 1D line, in contrast to 2D sheets of visual and somatosensory receptors, subsequent approximately topological station-to-station connections between nuclei in the auditory system have an ‘extra’ dimension across which to spread.

Despite the differences between somatosensory and visual maps (somatosensory cortical areas a ‘patchwork quilt’ compared to locally continuous visual areas), the receptor arrays in the visual and somatosensory system are both fundamentally arranged as *two*-dimensional surfaces. And in both systems, individual points on the sensory surfaces (retina or skin) are mapped to thin columns (‘lines’) by the axon terminal arbors that project to each subsequent station in the brain (e.g., different laminae in the dLGN, different cortical layers in V1) (see Figure 3).

Auditory system projections, however, typically exhibit characteristically different axon terminal geometry. This is because the auditory receptor array is essentially a *one*-dimensional line. At each point in the cochlea, there are three inner hair cells and one outer hair cell (Ashmore, 2008), with the latter serving as the main source of ascending tonotopic input. Along the spiral tonotopic axis of the cochlea, there is a long one-dimensional line of about 3,500 of these three-plus-one hair cell ‘points.’

As a result of this ‘dimensionality mismatch’ (1D sensory surface vs. 2D tangential cortical map), subsequent topological station-to-station connections between nuclei in the auditory system have an ‘extra’ dimension across which to spread. Individual auditory projection axon terminals often form what are sometimes called ‘lines’ in the auditory literature, but which actually have the geometry of gently curved two-dimensional sheets. Subsequent auditory projections often exhibit a similar ‘point-to-plane’ morphology that contrasts with the ‘point-to-line’ (point-to-column) morphology typical in the visual and somatosensory systems.

**FIGURE 4:**
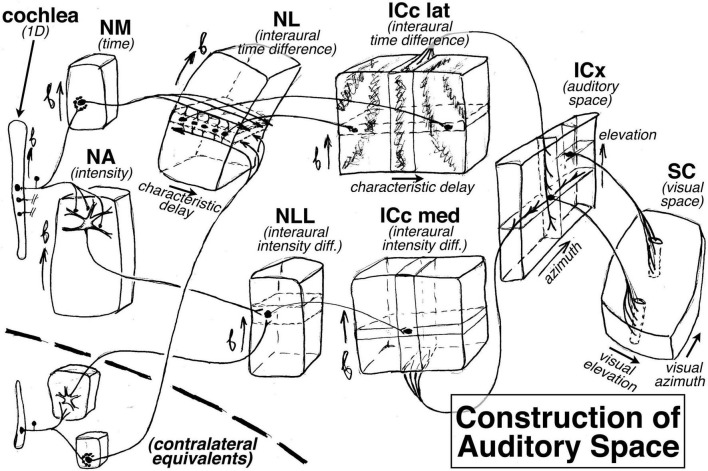
How the auditory system plays with maps: construction of an auditory space map from two (**left** and **right**) frequency maps in the barn owl. The ‘extra’ dimension perpendicular to tonotopy is used to construct maps of other features, such as characteristic delay in the nucleus laminaris (NL) and the inferior colliculus central nucleus lateral part (ICc lat), and eventually an auditory map of space in the external nucleus of the inferior colliculus (ICx), which is finally sent to the superior colliculus (SC).

The ‘extra’ dimension is put to good use in the auditory brainstem. For example, in barn owls (Konishi et al., 1988), there is a topological map of binaural characteristic delay that is oriented perpendicular to the tonotopic map in the nucleus laminaris (NL); it is constructed by arranging to have axonal delay lines come into the nucleus from the left and right monaural nucleus magnocellularis (NM) from opposite directions to synapse onto coincidence detecting neurons (see “NL” at upper left of Figure 4). Further along, a two-dimensional space map is constructed in the external nucleus of the inferior colliculus (ICx) by combining an interaural *time* difference map in the inferior colliculus, central nucleus, lateral part (ICc lat) with an interaural *amplitude* difference map in the inferior colliculus, central nucleus, medial part (ICc med, which signals elevation courtesy of the barn owl’s asymmetric ears); this is accomplished by intersecting arrays of point-to-plane projections from these two sources at right angles to each other in ICx (see “ICx” at the right side of Figure 4). Interestingly, this constructed auditory space map is then connected to the visual system (the superior colliculus) by what look like ‘standard-issue’ visual system point-to-line connections, in contrast to the point-to-plane style of connections in most of the previous stations of the auditory system. The mapping of characteristic delay in ICc lat probably has uses beyond the non-frequency-selective ICx space map. For example, by inhibiting all but one characteristic delay column in ICc, sounds coming from one particular azimuth could be isolated for further analysis by the thalamus and forebrain without destroying tonotopy (i.e., auditory spatial attention).

Finally, individual auditory areas in general have smaller surface areas than individual visual and somatosensory areas. This may be the result of the combination of a smaller total number of input receptors and the fact there is only one primary map dimension.

#### Comparing Phase-Encoded Maps Across Modalities

Given these substantial differences between maps in the three different modalities, we do not want to be too facile about directly comparing them. The patchwork quilt within each somatosensory cortical area differs from the more locally continuous representations in each cortical visual area. And for auditory areas, having only one primary map dimension makes finding borders perpendicular to the tonotopy gradient even more challenging; finally, what exactly is mapped in primate auditory cortex perpendicular to the tonotopic axis, if anything, remains disputed. In bats, it is known that there are higher level auditory areas that contain more complex two-dimensional auditory maps (e.g., the CF_*m*_/CF_*n*_ areas, where CF_*m*_ and CF_*n*_ refer to different harmonics of the constant frequency part of the outgoing call and echo); individual neurons there respond to particular pairs of frequencies and are arranged into two approximately orthogonal frequency gradients for the purpose of measuring Doppler shift independent of frequency (Suga, 1988), perhaps measured by sampling along diagonal lines across these two-frequency maps.

It is currently unknown whether something similar to the bat CF_*m*_/CF_*n*_ areas exist in human auditory areas (e.g., for identifying vowel formant patterns produced by speakers with different vocal tract sizes, a computationally similar problem to determining Doppler shift independent of outgoing frequency – in both cases, the spacing between two frequencies must be detected independent of absolute frequency); but in any case, our single bandpass stimuli would not have been optimal for revealing such a 2D map. In addition, because of constraints on scan and subject time, we were only able to map one dimension of retinotopy (polar angle, but not eccentricity); and in the somatosensory system, we have mapped one rough rostrocaudal ‘axis’ of main body parts from the toes to the face, without systematically interrogating the two spatial dimensions of the maps in each of these parts (e.g., in the case of the fingers, the palm to finger tip direction versus the perpendicular direction that runs from the radial to the ulnar side of each finger). Much longer experiments at higher resolution will be required to move forward from here.

### Visual, Somatosensory, and Auditory Maps in the Human Cortex

**FIGURE 5:**
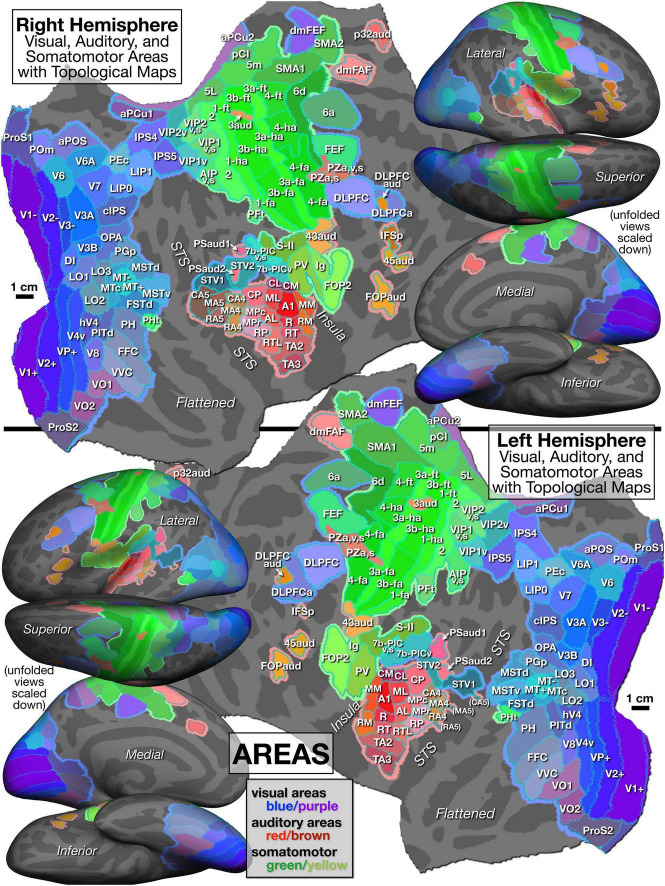
Parcellation of cortical areas containing topological sensorimotor maps as defined by significant amplitude response and significant phase spread to phase-encoded visual (blue/purple), auditory (red/brown), and somatomotor (green) mapping stimuli. See identically arranged Figure 6 for supporting mapping data and Table 1 for abbreviation definitions.

With all these caveats in mind, we divided cortex containing topological maps into 117 regions (57 visual, 34 auditory, 20 somatosensory, and 6 motor – see Table 1). These are illustrated in Figure 5, which displays the FreeSurfer annotation files: lh-CsurfMaps1.annot and rh-CsurfMaps1.annot (GIFTI versions are equivalent). To help perceptually group them, visual areas were colored with different shades of blue/purple, auditory areas colored red/brown, and somatomotor areas green/yellow. These areas occupied a little under half (47%) of the total surface area of the neocortex. In a few cases, large primary areas (e.g., V1, V2, V3/VP, MT, 3b, 1, 4) were subdivided into upper/lower field or hand/face/foot.

**TABLE 1:** Cortical area abbreviations by modality (annotation file order).

**Visual areas**	
*Primary, secondary, and tertiary areas*	
V1−	Striate cortex, lower field
V1+	Striate cortex, upper field
V2−	Second visual area, lower
V2+	Second visual area, upper
V3	Third visual area, lower
VP	Ventroposterior (=V3v)
*Lateral intermediate areas*	
DI	Dorso intermediate area
V3A	Visual area 3, accessory
V3B	Visual area 3, accessory B
OPA	Occipital place area
LO1	Lateral occipital area, 1
LO2	Lateral occipital area, 2
LO3	Lateral occipital area, 3
PGp	Parietal ang. area G, post.
*Posterior medial areas*	
V6	Visual area 6
V6A	Visual area 6 accessory
aPOS	Ant. parieto-occipital sulc.
POm	Parieto-occipital medial
ProS1	Area prostriata, 1
ProS2	Area prostriata, 2
*Lateral temporal areas*	
MT−	Middle temporal, lower
MT+	Middle temporal, upper
MTc	MT crescent (=V4t, DLa)
MSTd	Med sup. temporal, dorsal
MSTv	Med sup temporal ventral
FSTd	Fundus of STS, dorsal
STV1	Sup. temp. visual, area 1
STV2	Sup. temp. visual, area 2
7b-PICv	Area 7b parietal insular ctx
7b-PICv,s	Area 7b par. ins., vis/som
*Inferior intermediate areas*	
V4v	Visual area 4, ventral
hV4	Human V4
V8	Visual area 8
PITd	Post. inferotemp. dorsal
PH	Basal parietal area H
FFC	Fusiform face complex
VVC	Ventral visual complex
VO1	Ventral occipital area 1
VO2	Ventral occipital area 2
*Superior parietal areas*	
V7	Visual area 7
cIPS	Caudal intraparietal sulc.
LIP0	Lateral intraparietal zero
LIP1	Lateral intraparietal area
PEc	Parietal area E, caudal
IPS4	Intraparietal sulcus area 4
IPS5	Intraparietal sulcus area 5
aPCu1	Ant. pre-cuneus visual 1
aPCu2	Ant. pre-cuneus visual 2
*Superior parietal areas*	
VIP1v	Ventral intraparietal 1, vis
VIP1v,s	VIP1, visual and somato.
VIP2v	Ventral intraparietal 2, vis
VIP2v,s	VIP2, visual and somato.
*Frontal visual areas*	
dmFEF	Dorsomedial FEF
FEF	Frontal eye fields
6a	Area 6, part a
DLPFC	Dorsolateral prefontal ctx
DLPFCa	Dorsolateral PFC, part a
**Auditory areas**	
*Auditory core (primary)*	
A1	Primary auditory area
R	Rostral auditory area
RT	Rostro-temporal area
*Auditory caudal/medial belt (2°)*	
MM	Middle medial belt
RM	Rostromedial belt
CM	Caudomedial belt
*Auditory lateral belt (secondary)*	
CL	Caudolateral belt
ML	Middle lateral belt
AL	Anterior lateral belt
RTL	Rostrotemporal lateral belt
*Auditory para belt (tertiary)*	
CP	Caudal parabelt
MPc	Middle parabelt, caudal
MPr	Middle parabelt, rostral
RP	Rostral parabelt
TA2	Temporal area A, part 2
TA3	Temporal area A, part 3
*Auditory belt areas, 4th tier (A4)*	
CA4	Caudal 4th tier auditory
MA4	Middle 4th tier auditory
RA4	Rostral 4th tier auditory
*Auditory belt areas, 5th tier (A5)*	
CA5	Caudal 5th tier auditory
MA5	Middle 5th tier auditory
RA5	Rostral 5th tier auditory
*Subcentral area (tympanum)*	
43aud	Area 43, subcentral area
*Posterior sylvian areas*	
PSaud1	Posterior sylvian aud. 1
PSaud2	Posterior sylvian aud. 2
*Central sulcus auditory area*	
3aud	Area 3 auditory area
*Medial frontal auditory areas*	
dmFAF	Dorsomed front. aud field
p32aud	Area p32, auditory part
*Lateral frontal auditory areas*	
PZa,v,s	Polysensory zone, all 3
PZa,s	Polysensory zone, au/som
DLPFCaud	Dorsolateral PFC, aud.
IFSp	Infer. front. sulcus, post.
45aud	Area 45, auditory
FOPaud	Frontal operculum aud.
**Somatosensory areas**	
*Primary somatosensory areas*	
3b-fa	Area 3b, face and mouth
3b-ha	Area 3b, arm and hand
3b-ft	Area 3b, leg and foot
3a-fa	Area 3a, face and mouth
3a-ha	Area 3a, arm and hand
3a-ft	Area 3a, leg and foot
1-fa	Area 1, face and mouth
1-ha	Area 1, arm and hand
1-ft	Area 1, leg and foot
2	Area 2, face/hand/foot
*Higher somatosensory areas*	
5m	Area 5, medial
pCI	Post. cing. sulc., vis./som.
5L	Area 5, lateral
PFt	Parietal inf. F, tenuicortic.
AIPv,s	Anter. intrapar., vis./som.
*Lateral sulcus somatosensory areas*	
S-II	Secondary somatosensory
PV	Parietal ventral somato.
Ig	Insular granular field
FOP2	Frontal operculum, area 2
PHt	Bas. par. H, temporal entr.
**Motor areas**	
*Primary motor cortex*	
4-fa	Area 4, face and mouth
4-ha	Area 4, arm and hand
4-fo	Area 4, leg and foot
*Medial secondary motor areas*	
6d	Area 6, dorsal
SMA1	Supplementary mot. area
SMA2	Supplementary mot. area

*Visual areas* are grouped in Table 1 into primary, secondary and tertiary areas (e.g., V2), lateral intermediate areas (e.g., LO1), posterior medial areas (e.g., V6), lateral temporal areas (e.g., MT), inferior intermediate areas (e.g., V8), superior parietal areas including posterior cingulate areas (e.g., LIP1), and frontal visual areas (e.g., DLPFC). The lower-case suffixes are appended to multisensory areas (e.g., VIP1v,s) to indicate the modalities involved (e.g., visual plus somatosensory).

*Auditory areas* are divided into primary-like areas (e.g., A1), medial and lateral belt areas (secondary), lateral parabelt areas (tertiary), and fourth and fifth tier auditory belt areas (e.g., CA4, CA5 where “C” means caudal). Several other areas include the subcentral area (Brodmann 43) representing the tympanum (43aud), several frontal auditory areas (e.g., dmFAF), and a newly recognized central sulcus area (3aud) (see below for details).

*Somatosensory and motor areas* are divided into primary somatosensory and motor areas (e.g., 3b, 4), superior parietal areas (e.g., 5m), lateral secondary areas (e.g., S-II), and medial secondary motor areas (e.g., SMA1).

**FIGURE 6:**
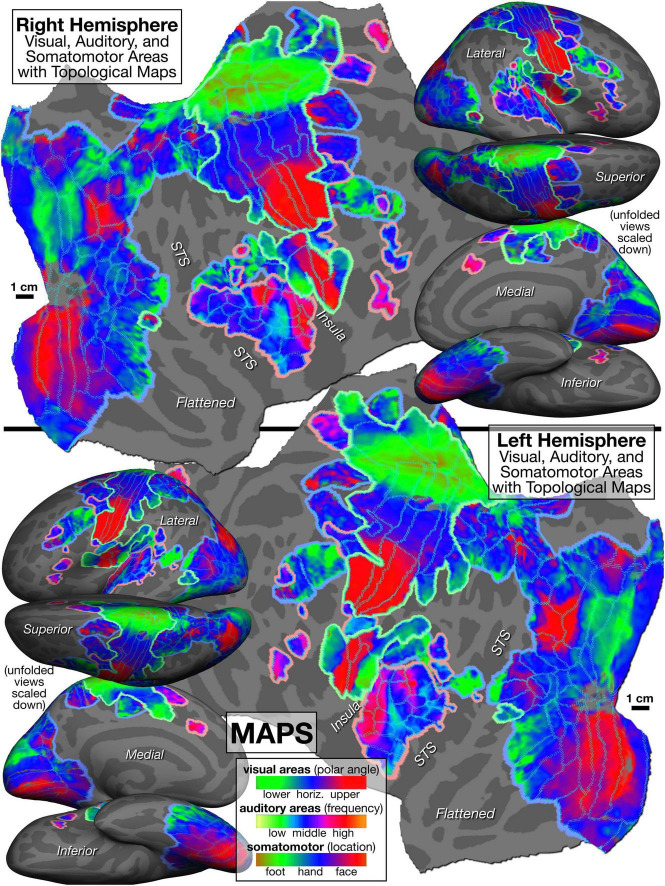
Topological cortical maps defined by periodic response to phase-encoded mapping stimuli (*visual*: clockwise/counter-clockwise rotating polar angle wedges; *auditory*: ascending/descending bandpass-filtered non-verbal vocalizations; *somatomotor*: face-to-foot/foot-to-face bilateral, cued voluntary movements of individual body parts). *Green* is lower field, low freq, or leg/foot; *blue* is horizontal meridian, mid freq, or arm/hand; *red* is upper field, high freq, or face.

The mapping data on which the Figure 5 parcellation is based is illustrated in Figure 6, with areal borders from Figure 5 superimposed using cyan dots. As introduced above, an intuitively similar color scale was used for each modality, where *green* indicates lower visual field, lower auditory frequency, or leg/foot; *blue* indicates horizontal meridian, middle frequency, or arm/hand; and *red* is upper field, high frequency, or face. Figures 5, 6 are sized and arranged identically to make it easy to blink back and forth between them in an image viewer (see also GIF animation of this^4^).

#### New Features of This Parcellation

For most of the areas in this new parcellation, there is no major dispute with the literature (beyond different author’s conflicting nomenclature, or minor differences in boundary placement). Several comments on new or unusual features follow.

In *retinotopic* maps in the visual system, area LIP1 is ‘putative human LIP’ as originally defined in Sereno et al. (2001). Parietal areas posterior and anterior to LIP1 were taken from the literature, respecting priority.

We outlined VIP1 and VIP2 using retinotopy, but then further distinguished their anterior halves (VIP1v,s and VIP2v,s), which have multisensory (visual plus somatomotor) maps (visual maps shown in Figure 6). However, since our coverage of the visual field was much better than our coverage of the body surface, it is likely that more complete stimulation of the skin surface would have extended the multisensory overlap somewhat posteriorly (see Huang et al., 2017, for a higher resolution investigation of multisensory overlap and variation in the VIP’s). We include both V4v and hV4 (sometimes contrasted), a third LO area (LO3), and another area adjoining V3A (OPA, occipital place area, Huang and Sereno, 2013). On the medial surface anterior to V6 and V6A and the periphery of V1 and V2, we define four areas; moving superior to inferior, these are: an anterior parietal-occipital sulcus area (aPOS), the medial parieto-occipital area (POm), and two retinotopic subdivisions of area prostriata (ProS1 and ProS2). Further anterior on the midline (after a small gap without a visual map) are two retinotopic anterior precuneus visual areas (aPCu1 and aPCu2). 7b-PIC in the posterior lateral sulcus is predominantly visual, but the anterior-superior part also has somatosensory input from the foot; these areas are distinguished as unimodal 7b-PICv and multisensory 7b-PICv,s.

In frontal cortex, we have labeled area CSv (cingulate sulcus visual area), an area anterior to the central sulcus, with its prior name from the non-human primate literature, dmFEF (dorsomedial frontal eye fields) (review: Tehovnik, 1995; also known as the supplementary eye fields).

In *tonotopic* maps in the auditory system, we placed the core areas A1, R, and RT along the highly myelinated crown (Dick et al., 2012) of Heschl’s gyrus with nearly parallel tonotopic gradient directions (see Leaver and Rauschecker, 2016, for a different proposed orientation of the auditory core relative to Heschl’s gyrus). We then defined four additional tiers of areas moving posteriorly and inferiorly from the core, using conventional abbreviations for the second and third tier areas (Kaas and Hackett, 2000). The second tier includes the medial (M) and lateral (L) belt, and the third tier is the parabelt (P), where within-belt subdivisions are indicated by a C/M/R prefix for caudal/middle/rostral. The fourth tier was named caudal/middle/rostral A4. The strong tonotopic responses in the right hemisphere in the fifth tier, caudal/middle/rostral A5 (CA5, MA5, and RA5), were weak in the left hemisphere. See Rauschecker and Scott (2009) and Rauschecker (2018) for a different, functionally-based definition of A4 and A5 by analogy with the functions of V4 and V5/MT as opposed to their distance from primary cortex; their A4 areas, by contrast, are the anterior auditory “what” stream, and their A5 areas, the posterior “how/action” stream. Finally, the most rostral temporal auditory area (TA2 and TA3) have higher numbers moving rostrally (following Jones and Burton, 1976, but in contrast to the foundational core-and-belt study of Pandya and Sanides, 1973, where Ts1 is the most rostral of Ts1, Ts2, and Ts3).

There was a small (2 mm) auditory/somatomotor overlap between a region at the border between the middle medial belt (MM) and the caudal medial belt (CM), and the foot representation in the parietal ventral somatosensory area (PV) (not subdivided here, auditory-somatosensory multisensory region included in PV).

Most unusually, we found a small bilateral superior central sulcus auditory area (low to high frequencies from anterior-lateral to posterior-medial) embedded within Brodmann area 3a/3b (3aud). It occupies about half of a small no-response gap in our somatomotor mapping data between the representation of the ‘hand’ (which includes the arm and stomach) and the ‘foot’ (which includes the thigh and leg) that likely corresponds to the representation of the genitalia (Knop et al., 2021) and/or respiratory muscles (McKay et al., 2003), both of which we did not specifically stimulate. Auditory responses in this region are not unprecedented (see Tierney et al., 2012, their Figure 2). We also describe a subcentral sulcus auditory area (43aud) lateral to the face S1/M1 face representation in Brodmann area 43 (low to high frequencies moving inferior to superior) (Job et al., 2011).

We outline seven tonotopic frontal auditory areas. Moving inferior to superior, these include two frontal opercular areas (FOPaud, 45aud), a small inferior frontal sulcus area (IFSp), a small area embedded within the anterior of two retinotopic dorsolateral prefrontal cortical areas (DLPFCaud), two tonotopic multisensory areas that intrude posteriorly into area 3a and 4, near the junction between the hand and face representations (PZa,s and PZa,v,s; both areas contain a face somatomotor representation, while the second also has a visual map; “PZ” is for polysensory zone, from Cooke and Graziano, 2004), a dorsomedial frontal auditory field that rounds onto the medial wall (dmFAF) adjoining the opposite side of the supplementary motor cortical areas from dmFEF, and a small auditory area further inferior within the cingulate sulcus (p32aud). Finally, there were two small posterior sylvian auditory regions (PSaud1,2) adjoining (in one case embedded within) superior temporal visual areas 1 and 2 (STV1,2).

In *somatomotor* maps, in the central sulcus and adjoining gyri, we subdivided areas 4, 3a, 3b, and 1 into “face,” “hand,” and “foot” (Kuehn et al., 2017; for finer subdivisions of the somatomotor “face” areas, see Carey et al., 2017). We distinguished five areas in the lateral sulcus moving posterior to anterior: a multisensory area in posterior insular cortex (7b-PICv,s), secondary somatosensory cortex (S-II), parietal ventral somatosensory (PV), insular granular (Ig), and a frontal opercular area (FOP2). Lateral to VIP1v,s and VIP1v, we illustrate a multisensory (visual and somatomotor) anterior intraparietal area (AIPv,s) containing a full body representation (Figure 6 shows somatosensory, not visual, representation), and further laterally, there is another hand and foot area, PFt (von Economo and Koskinas, 1925; Triarhou, 2007; the “PF” is for “parietal, inferior” and the “t” is for “tenuicortical”) that is posterior to the central sulcus face representations that protrudes posteriorly and inferiorly from area 1. Much further posteriorly, directly bordering temporal visual areas, there is a small somatosensory map, PHt (von Economo and Koskinas, 1925), at the anterior boundary between visual areas FSTd and PH (the posterior third of PHt also has visual responses, not subdivided here; the “PH” is for “parietal, basal” and this “t” is for “temporal entrance”). Finally, on the medial wall, between visual aPCu2 and somatosensory 5m, we delineated a visual plus somatomotor multisensory area, pCI (posterior cingulate sulcus multisensory area: Serra et al., 2019). Further anterior on the midline, we distinguish two supplementary motor areas joined at a representation of the upper body and face (SMA1 and SMA2).

#### An Atlas of Maps and Map Coordinate Gradients in Individual Areas

One of the difficulties that arises when averaging small topological maps across subjects is that slight map misalignments between subjects tend to compress the range of map coordinates within an area (‘regression toward the horizontal meridian’ in the visual system, ‘regression to mid frequencies’ in the auditory system), overemphasizing map coordinates in the middle of the maps. That can make it more difficult to discern the direction of the map coordinate gradient; the gradient can also be harder to see with partial representations of the sensory surface (e.g., visual hemifield versus quadrant versus other partial hemifield divisions – for the last case, see Gattass et al., 1988). Another set of difficulties for the reader’s visual understanding of these maps arises from just how many close-packed areas there are. This causes visual crowding from surrounding areas and partial map occlusion by border annotations (cyan border dots were used to allow some of the map data to show through).

Therefore, to provide a more user-friendly atlas for the new parcellation, we made a movie for the left and right flattened hemispheres (see Supplementary Videos 1, 2) that sequentially shows the mapping data from each of the 117 areas individually (234 Figures). We used a white arrow to indicate the average direction of the map coordinate gradient for each area, ∇ϕ(**r**), where ϕ(**r**) is the response phase at cortical position **r** (the gradient is the local steepest uphill direction in phase along the cortical surface; local phase gradient vectors are perpendicular to isophase lines). To annotate the map direction of the gradient, we used “lower/horiz/upper” for visual maps, “low f./high f.” (frequency) for auditory maps, and “foot/hand/face” for somatosensory maps. To aid interhemispheric comparison, we cropped the two movies and put them side by side (see Supplementary Video 3) so that the similarities in areal position, but especially, similarities in gradient direction across hemispheres (after mentally left-right mirror-imaging the gradient vector) were easier to appreciate. Each area is displayed for 1 s to make it easier to page through the atlas. Finally, the parcellation and map data have been displayed more traditionally on rotating folded surfaces (see Supplementary Video 4) and inflated surfaces (see Supplementary Video 5); the first rotations in each movie show the parcellation and the second, the map data (videos also at https://cogsci.ucsd.edu/~sereno/csurf/fsaverage-labels/CsurfMaps1-atlas-movies/).

### Maps/Cognition Overlap

The fraction of the cortex that contains topological maps (here approximately 47%) is larger than has usually been appreciated in the cognitive neuroscience literature. This suggests that areas with maps might be directly involved with many higher level ‘cognitive’ operations. To directly address the question as to whether areas containing topological maps participate in higher level ‘cognitive’ processes, we had subjects: (1) imagine navigating (eye closed) through familiar environments contrasted with imagining staying still (Huang and Sereno, 2013), or (2) naturalistically read short paragraphs contrasted with reading (same saccades) over paragraphs of unfamiliar (Hindi) characters (Sood and Sereno, 2016), or (3) have their gaze directed over scenes in a wordless picture story contrasted with the same saccade sequence over scrambled versions of the scenes (Sood and Sereno, 2018). In all three cases, well over half of all the ‘higher level’ activity (after the ‘low level’ subtractions) was situated in visual and auditory areas containing topological maps (see also Rauschecker and Scott, 2009). There was less overlap with non-eye movement related somatomotor areas; but perhaps this is not surprising given the general immobility of the body during the three ‘cognitive’ tasks.

### Maps in Action

The conditions under which we mapped topological areas – with unchanging central fixation for visual mapping, with eyes closed in the case of auditory stimuli, and with the great majority of the body virtually immobile for somatomotor mapping, is obviously highly artificial. For one thing, these simple mapping experiments conflate receptotopy and ‘attention-o-topy,’ which can be distinguished with more elaborate paradigms (e.g., for visual maps see Saygin and Sereno, 2008; for auditory maps see Dick et al., 2017). But in both of these cases (receptotopy and attention-o-topy) the maps are fixed. However, under even moderately more naturalistic conditions, a number of these maps have been shown to be dynamic and moveable.

#### Shifting Maps

For example, it is known that in area VIP, from both microelectrode experiments as well as fMRI experiments in humans (Avillac et al., 2005; Sereno and Huang, 2006) that the visual maps there are moveable relative to the cortex; the visual map is shifted using information from eye position to align the visual map with the essentially fixed-to-the-cortex somatosensory map of the face and axial body.

Movements of visual maps of a related kind have been found in area LIP (and a number of other parietal areas), where the retinotopic map (and much of its contents) temporarily moves just before a saccade to the position it will be in *after* the saccade (and then snaps back into alignment with the retina immediately after the saccade) using information from the deep superior colliculus about the planned eye movement (Duhamel et al., 1992; Heiser and Colby, 2006). This predictive movement may be preparing the system for how the world will look after the saccade; if nothing changes in the scene during the saccade, there will be no surprise at the new retinotopic map locations of all the stimuli after the saccade. These experiments in the cortex followed up on earlier experiments on double-step saccades in the superior colliculus that showed that the positions of salient targets are updated in retinotopic coordinates after each saccade so that even when a target has become invisible or camouflaged, the system is prepared to be able to make a saccade to its expected location (Mays and Sparks, 1980; Sparks and Hartwich-Young, 1989) by activating the corresponding location in the underlying saccade map in the deep colliculus (see below on the nature of the deeper map).

Auditory (spatial) maps can be shifted, too. As already noted above, experiments in the superior colliculus have shown that intermediate layer auditory spatial maps there, constructed from binaural comparisons, can be moved, despite a fixed head and thus a fixed interaural time delay and interaural amplitude difference, in order to dynamically keep the auditory spatial map in line with the retinotopic map that is being used to calculate which location in the deep colliculus saccade vector map should be activated for each successive saccade (Jay and Sparks, 1984).

Note that this auditory map shifting differs from the kind of map shifting that we previously described in VIP – auditory maps in the intermediate colliculus are moved to keep them in line with a fixed retinotopic map, while in VIP, retinotopic maps are moved to keep them in line with a fixed somatosensory face map. This difference reflects different goals. A major goal of the colliculus is to get the retinotopic *fovea* to a target (whether the target is visual or auditory or somatosensory) using the underlying *retinotopically*-organized saccade vector map; by contrast, a goal of VIP may be to avoid getting hit in the face by an object (or to help the face accurately bite a target), whether the target is somatosensory or visual, and independent of current retinotopic position.

#### Deep Cross-Modal Map Interactions

Multisensory interactions have often been studied in higher areas near the boundary between modalities, such as area VIP, situated at the border between visual and somatosensory cortex. Nevertheless, map-based multisensory interactions may be more ubiquitous than our simple division of areas into visual, auditory, and somatosensory would seem to imply. By touching one or another finger of a human subject with sand paper in the dark and then comparing somatotopic cortical responses (7T imaging) to responses from a second experiment where the same subjects simply viewed one or another finger of a hand being touched (in a first-person view), it was shown that merely observing touches results in a detectable amount of activity in the corresponding finger representation in primary somatosensory areas 3b and 1 (Kuehn et al., 2018). Remarkably, the visual signal somehow penetrated all the way back to the correct/corresponding finger representation in primary somatosensory cortex; and finger-specific, observation-related activation even generalized to an experiment using a third-person view of observed touch. Finally, there was evidence that the top-down observed-touch signal in areas 3b and 1 elicited activity in different cortical lamina outside those receiving direct ascending input from the ventrobasal nucleus (Kuehn and Sereno, 2018). The pathway by which the visual information got to area 3b and area 1 is currently obscure, but this data suggests that even low level maps can be ‘cognitive’ – or more precisely, single cortical columns within an area can contain map-coordinate-indexed information from markedly different levels of processing.

#### Traveling Waves and Bumps Across Maps

By applying the phase-encoded method to a more naturalistic reach-to-eat movement, it was possible to visualize the spatiotemporal unfolding of activity across map-containing areas. The unexpected result was that there was a spatially coherent traveling wave and bump activity that began in early visual areas and then swept over parietal cortex to the hand areas of somatomotor cortex eventually closing in on face somatomotor cortex (Chen et al., 2018). This is strongly reminiscent of similar (though much faster) coherent waves of activity visualized in the barrel cortex of awake rodents during active whisking using voltage sensitive dyes (Moldakarimov et al., 2018; see also Wu et al., 2008, who describe similar waves visualized in slices). This suggests that the numerical dominance of extremely local connections in the cortex illustrated in Figure 1 together with the presence of topological maps in the cortex more strongly shapes activity during naturalistic actions than is usually appreciated.

#### Motor Output Maps – Superior Colliculus and Frontal Eye Fields

Perhaps the best characterized motor output map is the retinotopically organized saccade vector map that is constructed in the deeper layers of the superior colliculus. The saccade vector generated by electrical stimulation at different 2D map locations in the deep colliculus results in a saccade appropriate to fixate a stimulus situated in the peripheral location of receptive field in the overlying superficial retinotopic map. The saccade vector is coded by the position of activity in the deep colliculus map, not by the strength of activity (or stimulation intensity) there. By contrast, the final motor output commands in the brainstem take the form of three positive-only Cartesian (lateral, up, and down) coordinates (firing rate variables to drive oculomotor muscles). Both the saccade burst pattern generators [*lateral*: paramedian pontine reticular formation (PPRF), *vertical* (*up, down*): rostral interstitial nucleus of the medial longitudinal fasciculus (riMLF)] as well as the oculomotor efference copy monitoring of eye position [*lateral*: prepositus hypoglossi, *vertical* (*up/down*): interstitial nucleus of Cajal] are coded by firing rate, *not* map position within these nuclei. The conversion from colliculus map position to firing rate is called the “spatio-temporal transformation” in the eye movement literature (with ‘temporal’ referring to firing rate). A simple way to implement it would be to have deep colliculus neurons from different map locations vary the strength of their connection to the horizontal and vertical (up and down) burst centers (Sereno, 1985; Sereno and Ulinski, 1985).

A similar kind of topological (retinotopic) saccade vector map is implemented in the frontal eye fields. The frontal eye fields have direct access to the horizontal and vertical saccade burst pattern generators in the brain stem (PPRF and riMLF), in parallel with the deep colliculus, and in communication with it.

But in order for efference copy eye position information to be able to shift 2D maps in the colliculus and cortex (as described above), these Cartesian firing-rate coordinates must somehow be converted back into positions within a 2D neural map. This is the much less well-understood “temporo-spatial” transformation. A possible implementation in the colliculus might involve axons from eye position centers synapsing across the colliculus in lines (e.g., see mediolateral-oriented tectal afferent axons from profundus mesencephali: Dacey and Ulinski, 1986b, their Figures 17, 18) contacting tectal cells with local axonal arbors that are systematically offset from the position of their dendritic arbors (e.g., see Dacey and Ulinski, 1986a, their Figures 7–9, 11–13; Sereno, 1985, their Figure 27). This shifting operation has been studied in the most detail in the intermediate and deep layers of the superior colliculus. But it must also be occurring independently in the cortex because brainstem-originating Cartesian eye position signals – that are known to transit through several thalamic nuclei (Tanaka, 2007) on the way to frontal oculomotor regions – have been shown to be critical for shifting retinotopic maps in posterior parietal cortex (LIP) during preemptive retinotopic map updating (Sommer and Wurtz, 2006). Finally, moveable spatial maps at many different levels in the brain must be coordinated; for example, the same eye position signals must somehow get to VIP in order to shift retinotopic maps into alignment with fixed somatotopic maps.

#### Motor Output Maps – Motor Cortex and Spinal Cord

Experiments in which multiple points were stimulated in the gray matter of the spinal cord in frogs have found that simultaneous stimulation of two sites results in the vector summation of the endpoint forces generated by simulating each site separately (Mussa-Ivaldi et al., 1994). More recently, similar results have been demonstrated in the primate spinal cord (Yaron et al., 2020). These results are quite reminiscent of what happens upon stimulating two sites in the deep colliculus, or one site in the deep colliculus and one site in the frontal eye fields (Schiller and Sandell, 1983), which both result in an eye movement that is a vector sum of the eye movements elicited by stimulating each site individually. The stability and repeatability of these spinal cord maps (Giszter et al., 2000) suggests that topological motor maps like the one in the deep colliculus may be the rule rather than the exception.

Finally, a series of experiments in which long stimulus trains were used in the motor cortex have suggested that topological maps of movement vectors are present there, too (see Aflalo and Graziano, 2007, for review and Kohonen model; see also Stepniewska et al., 2009, for similar results from stimulating primate posterior parietal cortex). The topological maps of movements in motor cortex are quite different than the patchwork quilt of skin regions described above in somatosensory cortical areas, though there is a rough match between main body part somatotopy in area 4 and that in areas 3a, 3b, and 1. In particular, these stimulation studies have found maps of nearby limb endpoints in particular regions of extrapersonal space. There are also different subregions that elicit different classes of ethologically relevant movements (e.g., acquisitive hand-to-mouth movements *versus* a defensive arm and hand movements). Because motor cortex controls a wider range of movements than the deep colliculus, it may consist of a patchwork quilt of multiple 2D vector maps. These results are broadly consistent with our finding of traveling waves and traveling bumps observed also in parietal and motor cortex in the phase-encoded reach-to-eat experiment (Chen et al., 2018).

## Conclusion and Speculation

The evidence presented above has concentrated on the half of the cortex that we have shown contains relatively straightforward visual, auditory, somatosensory, and motor maps. So what’s going on in the other half of cortex? Does it work entirely differently from the topologically mapped parts? We have seen that the strongly local connectivity found in areas with sensorimotor maps is also found in cortical areas without obvious maps. In early areas, there is no question that the exact spatial shape of an activation pattern across the cortex – e.g., activity due to a particular individual’s face – is crucial to recognition of that individual. A natural extension of this idea is that some of the apparently non-mapped regions may support moving, spatially localized disturbances with specific shapes similar to what regularly occurs in areas with maps, but detached from any fixed sensory map. We start by first drawing out the implications of the moveable maps reviewed above.

A number of the maps described were moveable, for example, as a mechanism of keeping maps from different modalities in register as the eyes move around relative to the head, body, and limbs. Map shifting has mostly been demonstrated to occur in areas that are several synapses beyond primary sensory areas (or in the case of the colliculus, in ‘higher level’ collicular layers that are anatomically below the direct retinal-recipient layers). Of course, in our phase-encoded mapping experiments, we were doing our best to *prevent* map shifting in order to be able to measure the coordinates of the maps under ‘neutral’ conditions (e.g., straight ahead gaze). Given how boring and long-drawn-out phase-encoded mapping paradigms are, it is possible that the apparently more ‘messy’ maps in Figure 6 in some of the higher areas could partly reflect poorer experimental control of map shifting in those areas.

It has generally been assumed that map shifting takes place within the bounds of a single cortical or subcortical area. In several cases, however, it is obvious that activity in maps can be shifted longer distances, namely, to the other hemisphere or other colliculus. For example, a brief target presented to the near right hemifield with central fixation will cause activity in the left colliculus. Before the eyes can move to it, a location in the left colliculus that causes a saccade to the far right hemifield is electrically stimulated. By using eye position information generated by electrical-stimulation-induced saccade, it is known that the original target will be shifted into the opposite colliculus from the one that originally viewed the target in order to generate the appropriate corrective saccade (Sparks, 1986). A similar target transfer to the opposite hemisphere has been demonstrated to occur in human LIP (Merriam et al., 2003), and can even occur when callosal connections have been severed (Berman et al., 2007).

Now re-consider the visualization of spatiotemporal activity during a naturalistic reach-to-eat task described above (Chen et al., 2018), which showed coherent traveling waves of activity sweeping across multiple topological maps, several of which have been demonstrated to be capable of map shifting. Given that the strong local connectivity present in the half of the cortex that contains maps is *also* present in the remaining half of the cortex without obvious sensory maps, this suggests an intriguing picture where spatially coherent activity might be able to ‘escape’ from sensorimotor maps and be injected into surrounding cortical areas that lack fixed maps. Since this activity would be even less tied to specific cortical locations than, say, visual activity in VIP that is being shifted around *within* VIP by eye position, it would be more difficult to visualize and average using standard low temporal resolution fMRI methods; however, it might be detectable with dense electrode arrays, dynamic optical imaging, or time-resolved echo-planar MRI (e.g., Wang et al., 2021).

But why might we want some soliton-like waves or localized attractor bumps (Wilson and Cowan, 1972; Zhang, 1996; Samsonovich and McNaughton, 1997; Baker and Cruz, 2021) to ‘escape?’ If we consider the computational problem of scene comprehension, it fundamentally involves a serial assembly process whereby information about different objects, actors, affordances, paths, and locations is built up over time for the purpose of controlling behavior. One can view other characteristically human cognitive operations such as language comprehension in a similar way – as code-directed serial assembly of fictive scenes (Sereno, 1991, 2014; Sood and Sereno, 2018). In both cases, there is the need to load chunks of isolated content – in the case of a scene, a glance at another monkey, at a branch, at a leaf, or in the case of language, a string of isolated word meanings – into working memory to allow these chunks to interact with each other. This process is often visualized as a buildup of persisting, spatially distributed activity.

Instead, we might think of each glance or word as initiating a localized cortical disturbance that ‘escapes’ into non-sensorimotor mapped cortex, and which can then be attached to other localized disturbances there to create a temporary, spatially extended ‘molecular’ pattern that is capable of propagating across parts of the cortical sheet while retaining its spatial topological structure. This initially might seem a strange picture; but it would not be out of place with the picture of spatially coherent, soliton-like traveling waves and bumps that we observed in during the reach-to-eat task, and with the partial overlap between cortex with maps and cortex involving in language and scene integration in temporal, parietal, and frontal cortex (Huang and Sereno, 2013; Sood and Sereno, 2016, 2018; Popham et al., 2021; Groen et al., 2022).

In the end, the goal would be to eventually inject some of that ‘escaped’ activity back into motor maps at various levels in order to manipulate arrays of muscles, or in the case of eye movements, to manipulate activity in the saccade vector map in the frontal eye fields and the deep layers of the superior colliculus, both of which require spatially localized bump shifting. Given the precedent of targets being able to be shifted into the opposite hemisphere, it might be possible to inject part of a temporarily ‘escaped’ pattern back into a spatially distant area in the cortex via corticocortical, cortico-thalamo-cortical, or other subcortical loop back connections. Earlier sensory maps represent objects and actions by the shape and movement of activation patterns there; rather than ‘maps all the way up,’ perhaps it’s ‘moving shapes all the way up.’

Existing theories of traveling waves or bumps typically start with a sheet with local excitation and slightly less local inhibition [both typically circularly symmetric and arranged similarly across the sheet (Wilson and Cowan, 1972; Zhang, 1996)]. With some form of input/output non-monotonicity (e.g., rapid habituation), traveling waves (rather than merely static bumps) can emerge. An early locally connected ‘neural network’ (cellular automaton) with a non-monotonic update rule is Conway’s Game of Life (too few neighbors → die, enough → birth/persist, too many → die). This simple architecture can nevertheless support myriad complex, composite propagating patterns of arbitrary sizes^5^ (“gliders,” “spaceships”). The idea above of building up moveable ‘molecular’ patterns that contain multiple localized attractor-like bumps that are temporarily bonded together is no more (or less) than an idea for a new theory of cortical computation. It goes beyond existing well-worked out theoretical frameworks, but it is a natural extension of how object shape, articulation, and actions are represented across early sensory and motor maps.

## Data Availability Statement

The datasets presented in this study can be found in online repositories here: https://cogsci.ucsd.edu/~sereno/csurf/fsaverage-labels and https://mri.sdsu.edu/sereno/csurf/fsaverage-labels.

## Ethics Statement

The studies involving human participants were reviewed and approved by UCSD Human Subject Committee, University College London Ethics Committee. The patients/participants provided their written informed consent to participate in this study. The animal study was reviewed and approved by Caltech Institutional Review Board.

## Author Contributions

MIS conceived of the study, wrote software, analyzed the data, made illustrations, and wrote the manuscript. MRS conceived of the study, collected data, wrote software, and analyzed the data. R-SH conceived of the study, constructed stimulus devices, wrote software, collected data, and analyzed the data. All authors contributed to the article and approved the submitted version.

## Conflict of Interest

The authors declare that the research was conducted in the absence of any commercial or financial relationships that could be construed as a potential conflict of interest.

## Publisher’s Note

All claims expressed in this article are solely those of the authors and do not necessarily represent those of their affiliated organizations, or those of the publisher, the editors and the reviewers. Any product that may be evaluated in this article, or claim that may be made by its manufacturer, is not guaranteed or endorsed by the publisher.

## Footnotes

1
https://cogsci.ucsd.edu/~sereno/mapper/

2
https://surfer.nmr.mgh.harvard.edu/

3
https://cogsci.ucsd.edu/~sereno/csurf/

4
https://cogsci.ucsd.edu/~sereno/csurf/fsaverage-labels/CsurfMaps1-illustrations/CsurfMaps1-areas,maps.gif

5
http://conwaylife.com

## Supplementary Material

Supplementary Video 1 The visual, auditory, and somatomotor maps in each of the 117 individual cortical areas in the parcellation are shown for 1 s each on the flattened left hemisphere of the FreeSurfer average subject, fsaverage. Major sulci are annotated at the beginning, and all areal borders are indicated by cyan dots. White arrows indicate the average gradient of the map coordinate for each area (“lower/horiz/upper” for visual maps, “low f./high f.” for auditory maps, and “foot/hand/face” for somatosensory maps). The color scale for the map data is: green for lower visual field, low frequency, or leg/foot; blue for horizontal meridian, mid frequency, or arm/hand, and red for upper field, high frequency, or face.

Supplementary Video 2 The visual, auditory, and somatomotor maps in each of the 117 individual cortical areas in the parcellation are shown on the flattened right hemisphere, using the same annotations and color scales as the left hemisphere (Supplementary Video 1).

Supplementary Video 3 The first two videos (Supplementary Videos 1, 2) havebeen cropped and juxtaposed so that similarities in areal position and gradient direction across the two hemispheres are easier to appreciate (after mentally left-right mirror-imaging them across a vertical axis).

Supplementary Video 4 The parcellation and map data are more conventionally displayed on the rotating folded surfaces of the left and right hemisphere of the FreeSurfer average subject, fsaverage (surface: white). The first rotation around a vertical axis shows the parcellation, and the second rotation shows the topological map data. After each vertical-axis rotation, the brain is rotated around a horizontal axis to expose superior and then inferior areas. The color scale for the map data is the same as in Figure 6 and Supplementary Video 1.

Supplementary Video 5 The parcellation and map data from Supplementary Video 4 are displayed on the rotating inflated surfaces of the left and right hemisphere of FreeSurfer average subject, fsaverage (surface: inflated_avg) in the same order, using the same color scales, and at the same magnification as in Supplementary Video 4.
